# Naturally acquired humoral and cellular immune responses to *Plasmodium vivax* merozoite surface protein 8 in patients with *P. vivax* infection

**DOI:** 10.1186/s12936-017-1837-5

**Published:** 2017-05-22

**Authors:** Yang Cheng, Bo Wang, Siriruk Changrob, Jin-Hee Han, Jetsumon Sattabongkot, Kwon-Soo Ha, Patchanee Chootong, Feng Lu, Jun Cao, Myat Htut Nyunt, Won Sun Park, Seok-Ho Hong, Chae Seung Lim, Takafumi Tsuboi, Eun-Taek Han

**Affiliations:** 1 Department of Medical Environmental Biology and Tropical Medicine, School of Medicine, Kangwon National University, Chuncheon, Gangwon-do 200-701 Republic of Korea; 2 Department of Parasitology, Wuxi Medical School, Jiangnan University, Wuxi, Jiangsu China; 3 Department of Clinical Laboratory, The First Affiliated Hospital of Anhui Medical University, Hefei, Anhui People’s Republic of China; 4 Department of Clinical Microbiology and Applied Technology, Faculty of Medical Technology, Mahidol University, Bangkok, 10700 Thailand; 5 Mahidol Vivax Research Unit, Faculty of Tropical Medicine, Mahidol University, Bangkok, 10400 Thailand; 6 Department of Molecular and Cellular Biochemistry, School of Medicine, Kangwon National University, Chuncheon, Gangwon-do 200-701 Republic of Korea; 7 Key Laboratory of Parasitic Disease Control and Prevention (Ministry of Health), and Jiangsu Provincial Key Laboratory of Parasite Molecular Biology, Jiangsu Institute of Parasitic Diseases, Wuxi, Jiangsu Province People’s Republic of China; 8 Department of Medical Research, Yangon, Myanmar; 9 Department of Physiology, School of Medicine, Kangwon National University, Chuncheon, Gangwon-do 200-701 Republic of Korea; 10 Department of Internal Medicine, School of Medicine, Kangwon National University, Chuncheon, Gangwon-do 200-701 Republic of Korea; 11 Department of Laboratory Medicine, College of Medicine, Korea University, Seoul, Republic of Korea; 12 Division of Malaria Research, Proteo-Science Center, Ehime University, Matsuyama, Ehime Japan

**Keywords:** *Plasmodium vivax*, Merozoite surface protein 8, Immunogenicity, Food vacuole

## Abstract

**Background:**

Thirty-one glycosylphosphatidylinositol (GPI)-anchored proteins of *Plasmodium vivax,* merozoite surface protein 1 (MSP1), MSP1 paralogue, MSP4, MSP5, MSP8, and MSP10 have been reported from homologs of *Plasmodium falciparum* by gene annotation with bioinformatics tools. These GPI-anchored proteins contain two epidermal growth factor (EGF)-like domains at its C-terminus. Here, *P. vivax* merozoite surface protein 8 (PvMSP8) are considered as potential targets of protective immunity.

**Methods:**

Recombinant PvMSP8 (rPvMSP8) was expressed, purified, and used for the assessment of humoral and cellular immune responses in *P. vivax*-infected patients and immune mice. Moreover, the target epitope of ant-PvMSP8 antibodies and subcellular localization of PvMSP8 was also determined.

**Results:**

The rPvMSP8 was successfully expressed and purified as soluble form as ~55 kDa. PvMSP8 was localized to the outer circle of pigments associated with the food vacuole. The rPvMSP8 protein had a high antigenicity (73.2% in sensitivity and 96.2% in specificity) in patients infected with *P. vivax*. IgG2 antibody subtype was the predominantly responses to this antigen. Antibody response to PvMSP8 increased up to day 7 and after that slightly decreased within a month. The longevity of anti-PvMSP8 antibody was stably sustained up to 12-year recovery patient samples. Most anti-PvMSP8 antibodies recognized two epitopes that were located outside the C-terminal EGF-like domain. The cellular immune response in *P. vivax*-exposed individuals produced high levels of IFN-γ and IL-10 upon PvMSP8 antigen stimulation in vitro.

**Conclusions:**

All data in this study suggest that PvMSP8 antigen has a potential to induce both humoral and cellular immune responses in patients with *P. vivax* infection. The subcellular localization of PvMSP8 confirmed that it was associated with the parasite food vacuole in blood-stage parasites. A further characterization of this protein will be useful for blood stage *P. vivax* vaccine development.

**Electronic supplementary material:**

The online version of this article (doi:10.1186/s12936-017-1837-5) contains supplementary material, which is available to authorized users.

## Background

The merozoite surface protein 8 (MSP8) is one of the glycosylphosphatidylinositol (GPI)-anchored proteins of blood-stage malaria parasites. It contains a signal sequence at N-terminus and two epidermal growth factor (EGF)-like domains at C-terminus with significant homology to those of *Plasmodium* species [1–4]. In previous reports, the immunization with full-length *Plasmodium yoelii* MSP8 (PyMSP8) fused with *P. yoelii* MSP1-19 induced MSP8-restricted T cell response and high and sustained levels of protective PyMSP1-19- and PyMSP8-specific antibody responses [5]. As a malaria vaccine candidate, the conserved, immunogenic T-cell epitope located in the C terminus of *Plasmodium falciparum* MSP8 (PfMSP8) (ΔAsn/Asp) is useful for protective efficacy, together with the fusion partner of poor immunogens such as PfMSP1-19/MSP8 (ΔAsn/Asp) [6]. The dominant B cells epitopes were mapped onto the C terminus of PfMSP8 antigen, which supports a high immunogenicity of PfMSP8 for further vaccine development [6]. However, another previous *P. falciparum* MSP8 knock-out study showed that it is not required for asexual stage parasite growth and replication [7]. Thus these results indicated that the immunogenicity of MSP8 may different from *Plasmodium* species.

PfMSP8 has been confirmed to localize to the parasitophorous vacuole (PV) of infected erythrocytes. Intriguingly, its C terminus is found in the food vacuole (FV) [8]. In blood-stage parasites, the parasite can be protected within the PV in erythrocytes, which are cells devoid of proteins, lipid biosynthesis, and intracellular compartments [9]. *Plasmodium* parasites internalize host cell haemoglobin, which is degraded in a specialized compartment, the FV [10]. Some characteristics of the FV, such as a low pH and the presence of proteolytic enzymes, may lead to its classification as a lysosome-like organelle [11]. This highly specialized organelle is present only in *Plasmodium* blood stages; in other words, it is absent from the mosquito and liver stages and is not found in other apicomplexan parasites. In addition to PfMSP8, the C terminus of another well-known GPI-anchored protein, PfMSP1, also constitutes the FV [12, 13]. The findings described above suggest that PfMSP8 plays a distinctive role in FV of infected erythrocytes.

However, as a homologue of PfMSP8, little is known about PvMSP8 as potential targets of protective immunity. Only one previous study showed the recognition of recombinant PvMSP8 with *Plasmodium vivax*-infected patients’ sera [14]. To propose PvMSP8 as vaccine candidate against blood stage *P. vivax* parasite, in this study, a high-level and long-lived immune response was observed against PvMSP8 in vivax malaria patients and a high immunogenicity was detected in rPvMSP8-immunized mice. Dominant epitopes were also mapped in the C terminus of PvMSP8 and its subcellular localization in the blood stage was shown to be at the FV.

## Methods

### Expression and purification of recombinant PvMSP8

Gene sequence of *Pvmsp8* was obtained from the PlasmoDB website (http://plasmodb.org; accession no. PVX_097625). Protein domains were further predicted using the Simple Modular Architecture Research Tool (SMART) (http://smart.embl-heidelberg.de/) [15, 16]. Recombinant PvMSP8 (rPvMSP8) with a truncated signal peptide (SP) and GPI anchor was expressed and purified using a wheat-germ cell-free (WGCF) expression system [17]. Briefly, the specific primers: PvMSP8F, 5′-GGGCGGATATCTCGAGGGAAACGTTAGCCCACCC-3′; and PvMSP8R, 5′-GCGGTACCCGGGATCCTTAGCAGTATATTCCGTCTCCCTCA-3′ were used for DNA amplification. Then, the PvMSP8 DNA was cloned into the pEU-E01-His-TEV-MCS vector (CellFree Sciences, Matsuyama, Japan). The rPvMSP8 protein was expressed using a WGCF expression system and purified using a Ni–NTA agarose column (Qiagen, Hilden, Germany), as described elsewhere [17]. The production of rPvMSP8 protein was separated using 12% SDS–PAGE and detected via Western Blot using an anti-Penta-His antibody (Qiagen).

### Immunization of mice with rPvMSP8

Female BALB/c mice, at 6–8 weeks of age (DaehanBiolink Co., Eumsung, Korea) were immunized with 20 μg of rPvMSP8 and phosphate buffered saline (PBS, pH 7.4) with complete Freund’s adjuvant (Sigma-Aldrich, St. Louis, MO, USA) using intraperitoneal route of administration. Three and 6 weeks after immunization, the equal volume of antigen with incomplete Freund’s adjuvant (Sigma-Aldrich) was boosted. Mouse blood samples were taken after the final booster injection, 2 weeks later. The antisera against PvMSP1 was also produced following the same procedure as PvMSP8 [17]. Animal experimental protocols were approved by the Kangwon National University, and the experiments were performed according to the Ethical Guidelines for Animal Experiments of Ehime University and Kangwon National University.

### Indirect immunofluorescence assay (IFA)

The schizont-stage-rich parasites of *P. vivax* were collected from malaria patients in Thailand, as described previously [18]. Briefly, the slides were blocked with PBS containing 5% nonfat milk, incubated with rabbit anti-MSP1-19 (1:200 dilution) [18] and mouse anti-MSP8 (1:100 dilution) as primary antibodies, followed by incubation with Alexa Flour 546-conjugated goat anti-rabbit IgG or Alexa Flour 488-conjugated goat anti-mouse IgG antibody (Invitrogen, Carlsbad, CA, USA) and nuclear staining with DAPI (Invitrogen). Then, the slides with ProLong Gold antifade reagent (Invitrogen) were mounted. The parasites were observed under oil immersion using a confocal laser scanning FV200 microscope (Olympus, Tokyo, Japan).

### Study sites and sample collections

One hundred and twelve serum samples from vivax-infected patients (mean age 25.5 years; range 18–42 years) from endemic countries and 80 samples from healthy individuals, assessed as being negative using microscopy, were collected in the Republic of Korea. The Myanmar samples (*n* = 56) were collected in 2012 from patients in Shwe Kyin area of Myanmar, and the Thai samples (*n* = 56) were collected from symptomatic, smear-positive patients from the Mae Sod district of western Thailand. For the 1-month follow-up study, 25 patients from the Shwe Kyin area were assessed at 7, 14, 21, and 28 days after treatment. The serum samples from healthy individuals with a vivax malaria history were collected from Chinese residents who had an episode of vivax malaria infection in malaria endemic areas of Anhui Province, China, in 2012, and who did not have a reinfection episode of vivax malaria in the preceding 5 (*n* = 30), 12 (*n* = 30), or 30 (*n* = 30) years (Table 1).

**Table 1:** Characteristics of study *Plasmodium vivax* samples from endemic areas of Korea, Myanmar, Thailand and China

Characteristic	Value							
	Acute vivax patients			1 month	Recovery subjects (China)			Healthy subjects (Korea)
	Korea	Myanmar	Thailand	follow-up	5 year	12 year	30 year	
Total (*n*)	112	56	56	25	30	30	30	80
Age (year)								
Mean (SD)	25.5 (9.50)	23.3 (8.50)	30.1 (12.46)	21.6 (7.85)	52.5 (22.52)	49.3 (16.47)	62 (13.0)	9.5 (3.30)
Range	4–79	8–52	16–70	14–45	7–85	19–78	40–84	6–13
Parasitaemia (%)								
Mean (SD)	0.128 (0.11)	0.082 (0.11)	0.130 (0.12)					
Range	0.012–0.45	0.009–0.801	0.006–0.400					

For the cellular immunity study, 10 mL of heparinized blood from *P. vivax* subjects (*n* = 15) who had recovered from *P. vivax* infection after 8–10 weeks were collected at malaria clinics in Tha Sae, Chumphon Province, which is located in the southern peninsular region of Thailand. Moreover, heparinized blood from healthy individuals (*n* = 15) who had no history of exposure to malaria were collected. The samples were used for peripheral blood mononuclear cell (PBMC) preparation.

### Humoral immune responses and IgG isotyping

Amine (NH_2_-)-coated slides were prepared as previously described [19]. Serum samples from 112 cases of vivax malaria and 80 healthy individuals were used for humoral immune response analysis via well-type amine arrays. The chips were probed, scanned, and analysed as described previously [19]. The prevalence of an IgG isotype specific to PvMSP8 in the sera of 50 vivax patients and 10 healthy individuals was evaluated. Briefly, rPvMSP8 (50 μg/mL) was used for coating, followed by blocking and addition of human sera. Then these coated proteins were incubated with each IgG isotype for isotyping assay. The reaction was detected and analysed as described above.

### Epitope mapping using a peptide array

An array of 22 peptides, 18-mer each (Additional file 1: Table S1) overlapping by nine amino acids with >90% purity and spanning the conserved C terminus of PvMSP8 Sal-1 sequences was custom synthesized, purified, and used for epitope mapping (Peptron Co., Ltd., Daejeon, Korea). In the process of coating step, 1 μL of peptides (10–40 μg/mL) in 200 mM 1-ethyl-3-(3-dimethylaminopropyl) carbodiimide (EDC, Thermo Fisher Scientific Inc., Rockford, IL, USA) and 50 mM N-hydroxysuccinimide (NHS, Thermo Fisher Scientific Inc.) with coupling buffer were coated on amine-coated slide, respectively. After blocking, rPvMSP8-immunized mouse sera, pre-immunized mouse sera at 1:200 dilution, pooled sera from 10 high IgG titers of vivax-infected patients at 1:50 dilution, or 10 vivax-unexposed human sera at 1:50 dilution was added, respectively. Alexa Fluor 546 goat anti-mouse IgG (50 μg/mL, Invitrogen) or Alexa Fluor 546 goat anti-human IgG (10 μg/mL, Invitrogen) antibodies were used for detection of binding activity.

### Naturally acquired cellular immunity in *P. vivax*-exposed individuals

Lymphocyte stimulation assay was carried out for measuring of cellular immunity in 15 recovered *P. vivax* subjects. Briefly, 2.5 × 10^5^ PBMCs/well were stimulated with 10 μg/mL of rPvMSP8, 2% v/v of PHA, or complete RPMI 1640 medium only. After 96 h of stimulation, cytokine levels in the lymphocyte culture supernatant were measured using a BD OptEIA kit (BD Biosciences, San Jose, CA, USA). The phenotypes of T cells that responded to rPvMSP8 were analysed by intracellular cytokine staining after in vitro stimulation with 10 μg/mL of rPvMSP8, 20 ng/mL of PMA/ionomycin, or medium alone. Cells were stained with monoclonal antibodies (mAbs) against surface determinants of CD3, CD4, and CD8 and intracellular cytokine markers of anti-IFN-γ and anti-IL-10 antibodies, according to the manufacturer’s instructions.

### Cellular immunity in PvMSP8-immunized mice

A splenocyte proliferation assay was performed using splenocytes removed from mice that had been immunized with rPvMSP8. The cells were stimulated with rPvMSP8 (2.5 μg/mL), Con A (5 μg/mL), LPS (10 μg/mL), or medium alone. Culture supernatants were collected after 72 h of incubation and assayed using a BD CBA Flex Set kit (BD Biosciences), according to the manufacturer’s instructions.

### Statistical analysis

The correlation between the antibody reactivity of different concentrations of the recombinant proteins and duplicate spots of protein arrays was observed using GraphPad Prism software, version 5.0 (GraphPad, San Diego, CA, USA) and PASW Statistics 18.0 (SPSS Inc., Chicago, IL, USA). Sensitivity and specificity were measured by the percentage of patients who had a positive test result and the percentage of healthy individuals who had a negative test result, respectively. The significance of differences in mean fluorescence intensity (MFI) values between every two groups were performed with the Mann–Whitney U test.

## Results

### Structure, expression, purification, and western blot analysis of rPvMSP8

**Fig. 1:**
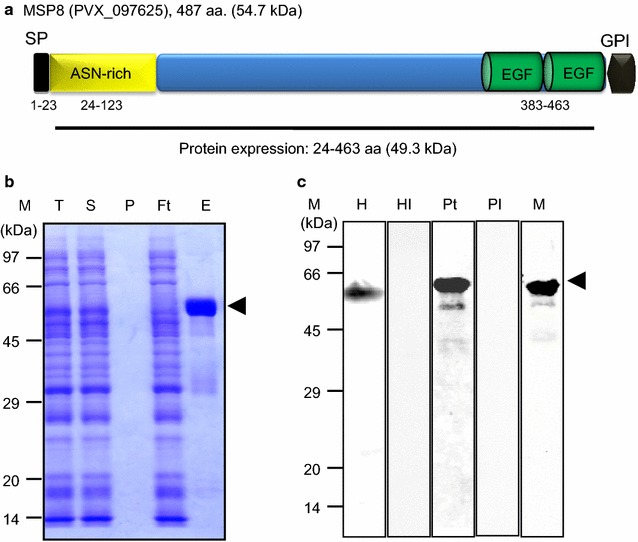
Schematic diagram, recombinant protein expression, purification, and western blot analysis of rPvMSP8. **a** Schematic diagram of PvMSP8. The PvMSP8 protein comprises 487 amino acids, with a calculated molecular mass of 54.7 kDa. The GPI anchor (amino acid [aa] position 464–486), the EGF domain (aa 383–463), the asparagine-rich region (ASN, aa 25–123), and the signal peptide (aa 1–23) are indicated. A truncated PvMSP8 (aa 24–463) was constructed for expression. T, total; S, soluble; P, pellet; Ft, flow-through; E, elution. **b** The purification progress of rPvMSP8 (55 kDa) was resolved by 12.5% SDS-PAGE. **c** Western blot analysis of rPvMSP8 using an anti-Penta-His antibody (H), healthy individuals (HI), mixed vivax patient sera (Pt), pre-immune sera (PI), and mouse immune sera (M), respectively. *Arrowheads* indicate specific bands for each recombinant protein. M, molecular size marker

The predicted molecular weight of PvMSP8 (PVX_097625) is 54.7 kDa, with 487 amino acids containing a signal peptide, asparagine-rich region in N-terminal region and two EGF domains and GPI-motif in C-terminal region (Fig. 1a). The recombinant protein encoding the truncated PvMSP8 (ΔSP/GPI) was successfully expressed and purified as soluble form using a WGCF expression system. The purity of the purified recombinant PvMSP8 proteins were assessed, and the protein migrated as a single band of 55 kDa from SDS-PAGE analysis (Fig. 1b). The corresponding immunoblots probed with an anti-His tag antibody, anti-rPvMSP8 immune mouse serum, or a mixture of vivax patient sera revealed a similar and specific pattern of migration for PvMSP8 (Fig. 1c), whereas preimmune mouse sera and healthy individuals serum samples in non-endemic areas were used as negative controls; recombinant Pv41 did not react with either reagent.

### Subcellular localization of PvMSP8

**Fig. 2:**
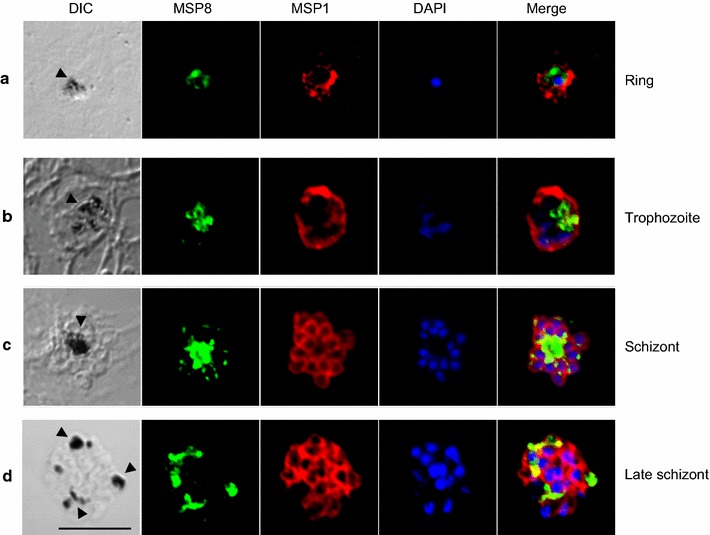
Subcellular localization of PvMSP8. Parasites were double-labeled with antisera against PvMSP8 and either PvMSP1-19 (merozoite surface marker) in the ring stage **a**, late trophozoite stage **b**, or schizont and late-schizont stages (**c**, **d**). Nuclei are visualized with DAPI in merged images. The *bar* represents 5 μm

To determine PvMSP8 localization, IFA was carried out using anti-PvMSP8 and anti-PvMSP1-19 immune sera. In blood-stage parasites of *P. vivax*, PvMSP8 was localized to the outer circle of pigments associated with the FV (Fig. 2, arrow heads). A comparison with PvMSP1-19 in different intraerythrocytic-stage parasites indicated that PvMSP8 localized on pigments densely from the ring to late trophozoite stage (Fig. 2a, b) and on scattered pigments from mature to late-schizont-stage parasites (Fig. 2c, d).

### Humoral immune response analysis of PvMSP8 in vivax malaria patients

**Fig. 3:**
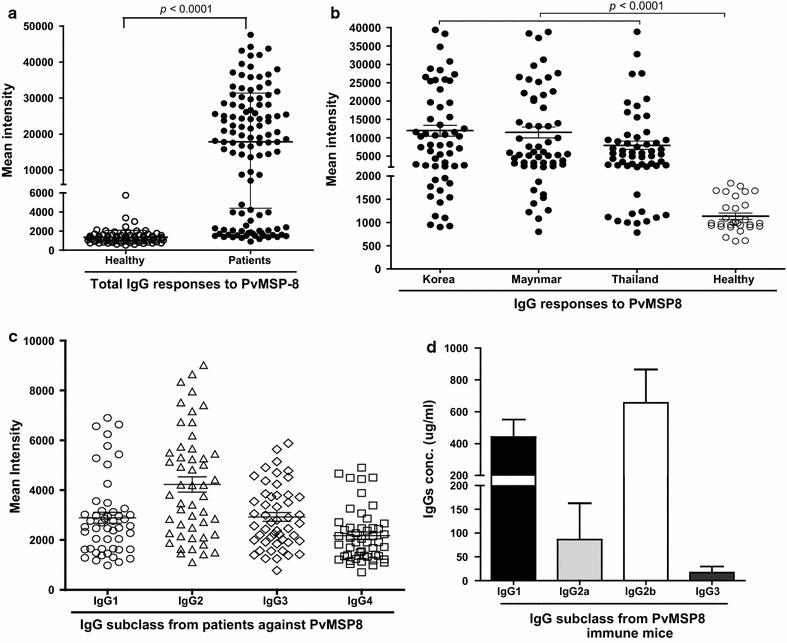
Total IgG and IgG subclass responses to rPvMSP8. **a** rPvMSP8 was probed with the sera of 112 malaria patients and 80 healthy individuals from the ROK. Significant differences were observed between vivax patients and healthy individuals in the total prevalence of anti-rPvMSP8 IgG (*P* < 0.0001). **b** Comparison of the IgG responses to PvMSP8 among the Korea, Myanmar and Thailand. Significant differences were observed between vivax patients from Korea, Myanmar and Thailand and healthy individuals in the IgG prevalence of anti-rPvMSP8 IgG (*P* < 0.0001). **c** The immunoreactivity of IgG subclass antibodies against rPvMSP8 using the sera of 50 patients randomly selected from those described above was also determined. The cutoff value was defined as two standard deviations (SDs) above the mean fluorescence intensity of eight negative control samples. The *error bars* indicate the mean ± SD. **d** IgG subclass levels in immune mice. The results are expressed as mean concentrations ± SDs

Antibody responses against rPvMSP8 in serum samples from 112 *P. vivax*-infected patients and 80 healthy individuals were determined. Seropositivity of anti-rPvMSP8 antibodies was 73.2% in sensitivity and 96.2% in specificity (Additional file 1: Table S2). These sera from *P. vivax*-exposed individuals exhibited a significantly higher MFI than did those from malaria-naïve subjects (Fig. 3a, *P* < 0.0001). Furthermore, PvMSP8 produced similar IgG titers among isolates of vivax malaria patients from Korea, Myanmar, and Thailand, as well as from malaria-naïve individuals (Fig. 3b). The sera of *P. vivax*-exposed individuals had a significantly higher MFI than sera from malaria-naïve subjects.

### Prevalence of IgG subclass response against PvMSP8 among malaria patients and immune mice

Protective antibodies against blood-stage parasites have been shown to belong to cytophilic classes; hence, the IgG subclass distribution was analysed. The prevalence of specific IgG subclasses was extremely high: >80% of the individuals had IgG antibodies that reacted with rPvMSP8 (Fig. 3c). Antibody responses against rPvMSP8 in vivax malaria patients were predominantly non-cytophilic IgG2 responses, which indicated that the efficacy of their protection may be poor against blood-stage parasites of *P. vivax*.

The isotypic distribution of anti-rPvMSP8 antibodies from immunized mice was analysed, assuming that the cytophilic IgG2a and IgG2b mouse isotypes would correspond to a Th1 response, whereas the non-cytophilic IgG1 and IgG3 would correspond to a Th2 response (Fig. 3d). Cytophilic antibodies (IgG2a and IgG2b) against PvMSP8 were the major components of the antibody response, especially IgG2b, observed in immunized mice. Non-cytophilic IgG antibodies were predominant in patients however cytophilic IgG antibodies were predominant in immune mice.

### Longitudinal analysis of the IgG immune response against PvMSP8

**Fig. 4:**
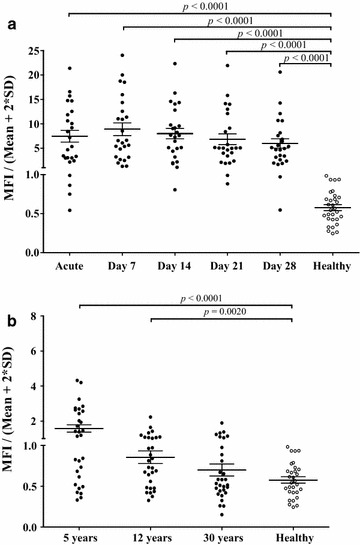
IgG antibody responses to rPvMSP8 in the sera of malaria patients followed for 1 month, archival vivax malaria patients (5-, 12-, and 30-year recovery), and malaria-naive individuals (Healthy). **a** IgG response of rPvMSP8 to 25 1-month follow-up vivax samples. **b** IgG response of rPvMSP8 to the sera of 5-, 12-, and 30-year archival vivax patients. The *vertical axis* (MFI/Mean ± 2SD) represents the mean fluorescence intensity divided by the mean fluorescence intensity plus 2 standard deviations of the malaria-naive samples. The *horizontal bars* indicate the means ± SDs. The Mann–Whitney *U* test was used for statistical analysis and *P* values calculation

The immune reactivity of the antibody response from serum samples during the 1-month follow-up after treatment in 25 patients with acute vivax malaria was assessed. Antibody response to PvMSP8, when detected, generally increased between the day of presentation, peaking on day 7, and was followed by a slight decrease from days 7 to 28. However, no significant difference was observed regarding IgG titers within 1 month (Fig. 4a; Additional file 1: Table S3).

The longevity of anti-PvMSP8 antibody responses was also analysed using long-term after exposure to vivax parasites (5-year recovery, 12-year recovery, and 30-year recovery samples) from China. A significantly higher IgG reactivity was observed in 5- and 12-year recovery sera, although the IgG titer was lower than that observed for acute infection. Moreover, in most cases, IgG antibody levels in individuals who infected malaria 30 years previously had decreased to baseline levels (Fig. 4b; Additional file 1: Table S3). These results indicate that IgG antibody responses against PvMSP8 are stably sustained in this population.

### Mapping of the dominant epitopes of the PvMSP8 C terminus

**Fig. 5:**
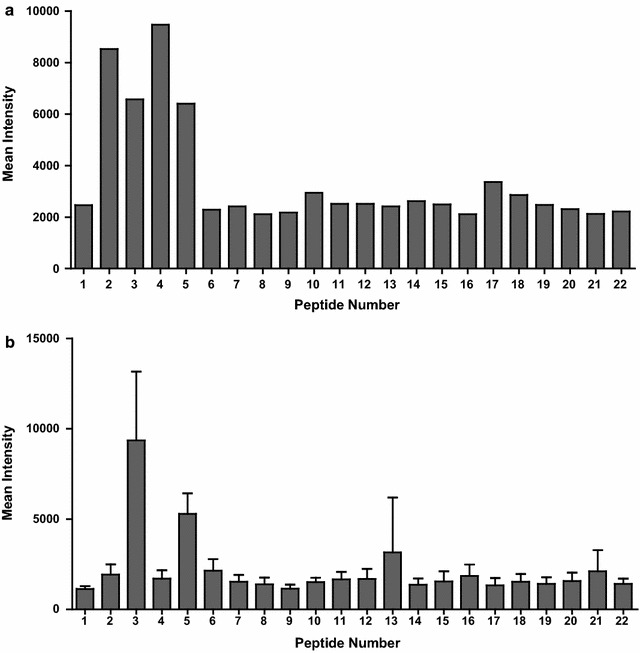
Reactivity of human and immunized mouse sera to an overlapping PvMSP8 conserved C-terminal peptide array. The average fluorescence intensity is shown for the peptides with pooled sera from vivax-infected patients (*n* = 10) **a** or with rPvMSP8-immunized mouse sera **b**. The *error bars* represent the geometric means of groups of mice ± SDs

A peptide was considered part of the potential epitope that there was significantly greater antibody reactivity for the antibodies in the vivax patient group compared with the vivax-unexposed human group, or greater reactivity for antibodies in rPvMSP8-immunized mice compared with preimmunized mice. Based on the reactivity of the pooled sera from vivax-infected patients, four peptides (nos. 2–5) located in the N terminus were highly reactive (Fig. 5a); based on those of immune mouse sera, two peptides (nos. 3 and 5) located in the central region and one peptide (no. 13) located in the C-terminal part were also highly reacted (Fig. 5b). Intriguingly, the two groups of sera were highly reacted with two peptides (nos. 3 and 5) located in the central region of PvMSP8. As a negative control, antibodies from preimmunized mice or unexposed human individuals that reacted with individual peptides were defined (all intensity values <1500).

### Cellular immune response against rPvMSP8

**Fig. 6:**
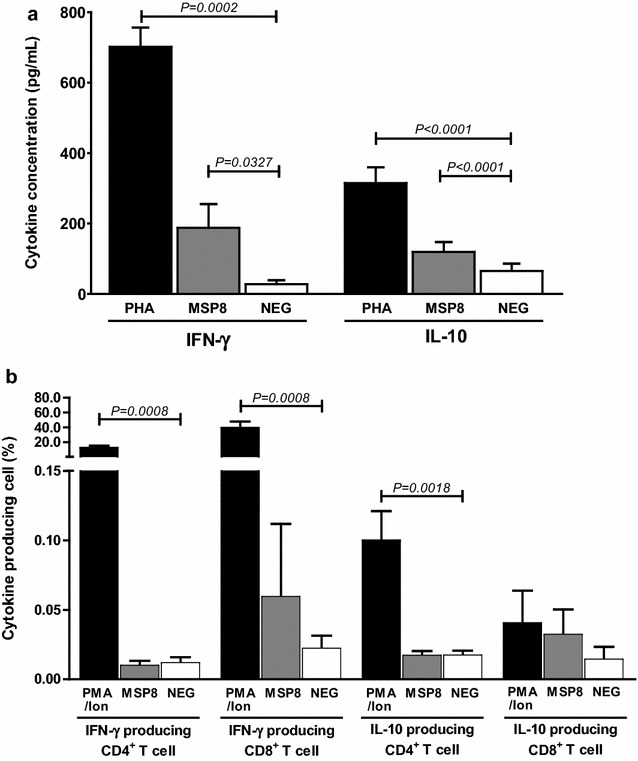
Cytokine response to the rPvMSP8 antigen. PBMCs from individuals that exhibited recovery from *P. vivax* infection for 8–10 weeks (*n* = 15) were stimulated with rPvMSP8. PHA and medium culture were the positive and negative control in each experiment, respectively. **a** The levels of the IFN-γ and IL-10 cytokines were detected in 96-h culture supernatants by ELISA. The data show the average value of cytokine levels. **b** IFN-γ-producing cells and IL-10-producing cells respond to PvMSP8 antigen were identified via intracellular staining after 6 h of in vitro stimulation and analysed using flow cytometry. PMA/ionomycin and medium culture were the positive and negative control in each experiment, respectively. Significance was determined using the Wilcoxon signed-rank test, with the level of significance set at *P* < 0.05

The determination of IFN-γ or IL-10 cytokine levels after in vitro rPvMSP8 stimulation showed that IFN-γ was significantly upregulated (fourfold) in comparison to that observed in the absence of antigen stimulation (rPvMSP8 = 188.10 pg/mL, no stimulation = 27.68 pg/mL, *P* < 0.05; Fig. 6a). Moreover, the levels of IL-10 in rPvMSP8 cultures were significantly higher (twofold) in comparison to that observed in the absence of stimulation (rPvMSP8 = 119.17 pg/mL, no stimulation = 65.44 pg/mL, *P* < 0.05; Fig. 6a). The PBMCs from *P. vivax* subjects produced higher levels of IFN-γ and IL-10 in response to PHA and rPvMSP8 than did those of the healthy controls. The phenotyping of IFN-γ- or IL-10-producing cells that responded to the PvMSP8 antigen showed that CD8^+^ T cells were IFN-γ-producing cells; they produced IFN-γ at levels three times higher than those observed in un-stimulation (rPvMSP8 = 0.059%, no stimulation = 0.022%; Fig. 6b). In contrast, IFN-γ levels were low in CD4^+^ T cells (rPvMSP8 = 0.010%, no stimulation = 0.012%; Fig. 6b). Neither CD4^+^ nor CD8^+^ T cells produced IL-10 cytokine in response to the rPvMSP8 antigen (Fig. 6b). The cytokine responses from rPvMSP8-immunized mouse splenocytes stimulated by various concentrations of the rPvMSP8 protein, ConA (positive control), LPS (positive control), and culture medium alone (negative control) (Additional file 1: Figure S1A), respectively, showed predominant IFN-γ and TNF secretion compared with IL-2, IL-10, and IL-4 levels (Additional file 1: Figure S1B).

## Discussion

PvMSP8 is a conserved GPI-anchored antigen that has been explored as potential antigens for vivax malaria vaccine development [4, 19]. As potent immune responses in both humans and mouse models were induced, PvMSP8 might be the target of actively induced B- and T-cell immune responses, which underscores its vaccine candidate potential.

In previous study about PvMSP8 [14], it could not defined exact subcellular localizations of blood-stage parasites with anti-PvMSP8 peptide antibody. However, in this study, the exact location of PvMSP8 was demonstrated that its FV subcellular localization surrounding pigments from intraerythrocytic-stage parasites were confirmed; therefore, PvMSP8 was referred to as an FV membrane-associated protein. In *P. falciparum* parasites, PfMSP8 has been shown to appear to co-localize on plasma membranes with PfMSP1, and only on the surface of early-ring-stage parasites, and its C terminus was found in the FV of infected erythrocytes in schizont-stage parasites [8]. In *P. yoelii* parasites, PyMSP8 was detected on ring-stage parasites and expressed together with PyMSP1 on the surface of each merozoite of mature schizont-stage parasites. In addition, PyMSP8 was highly expressed on the FV of trophozoite- and schizont-stage parasites [20]. However, the IFA images, which were produced using SP- and GPI-truncated PvMSP8 antibodies, showed that PvMSP8 (Fig. 2) was mainly expressed around the FV. Although weak reactivity of PvMSP8 was detected at the merozoite surface in schizont-stage parasites, the localization of PvMSP8 was obviously different from that of PvMSP1, which suggests different roles for the two proteins.

In previous studies, non-cytophilic classes (IgG2) also predominated among the anti-malarial antibodies developed by unprotected subjects, whereas cytophilic subclasses (IgG1 and mainly IgG3) were the most abundant isotypes produced by malaria patients, who are protected from malarial parasites [21, 22]. Conversely, a recent study showed an association between parasite levels and antigen-specific IgG2 and resistance to *P. falciparum* infection, suggesting that IgG2 plays a noncytophilic isotype role and contributes to parasite clearance [23]. In addition, purified IgG2 antibodies have been shown to block the ability to inhibit parasitic growth in vitro study. IgG2 responses were also related with the higher risk for severity of malarial infection in Kenyan children [24]. In this study, the high IgG2 response against PvMSP8 antigen was found in vivax patients, suggesting that PvMSP8 induce antibody production in patients, which may be associated with resistance to vivax malaria.

Here, the authors also observed that long-term maintenance of IgG antibodies against PvMSP8 was detected in individuals from Anhui Province, China (where malaria is not endemic recently, Fig. 4). Because the half-life of the human IgG molecule is around 21 days [25], the long-term maintenance of IgG antibodies may contribute to ongoing secretion of antibodies from plasma cells or to memory B cell differentiation in response to inflammatory stimuli. To investigate the actual longevity of antibodies against the PvMSP8 protein, a future longitudinal field study is required.

For vaccine consideration, conserved regions should include immunodominant protective T- and/or B-cell epitopes [26]. It appears that the immunogenic peptides involved in T-cell response are located at the C terminus of PfMSP8 [6]. To examine further B-cell epitopes, the antigenicity of each peptide was also compared. Both vivax patient serum and PvMSP8-immunized mouse serum antibodies were highly reacted with two peptides located outside the C-terminal EGF-like domain (Fig. 5). These data suggest that rPvMSP8 is highly immunogenic for both B and T cells, and that the C terminus appears to contain the dominant B-cell epitopes of PvMSP8. However, it remained to be further studies of potential vaccine candidates from B- and T-cell epitopes of PvMSP8.

As cytokine play an important role in cell-mediated immunity, the study showed that PvMSP8 stimulated PBMCs to produce the IFN-γ and IL-10 cytokines (with the IFN-γ-type response being more pronounced), and that CD8^+^ T cells were a major type of IFN-γ-producing cells (Fig. 6a, b). This indicates the immunogenicity of PvMSP8 in the induction of a cellular response against *P. vivax* parasite, which has been shown to be associated with protection against *P. falciparum* among volunteers undergoing experimentally induced infection [27], as well as in naturally exposed human populations [28, 29]. CD8^+^ T cells exhibited a stronger participation in the response to PvMSP8 than CD4^+^ T cells, whereas other IFN-γ-producing cells, including γδ T cells, NKT cells, and NK cells, should be considered in future studies. The regulation of the immune response in PvMSP8-stimulated PBMC cultures may be involved in IL-10 secretion, as it occurred significantly in the supernatant of 96 h cultures, whereas no evidence of immediate production of IL-10 by CD4^+^ and CD8^+^ effector cells was found upon short-term in vitro PvMSP8 stimulation.

## Conclusions

The study results demonstrated the presence of PvMSP8-antigen-induced humoral and cellular immune responses in *P. vivax* infection and represent an important advance in the understanding of blood-stage immunity to *P. vivax*, at least in part. Intriguingly, the manner via which a potent immune response, such as the one shown here, can be induced remains to be studied, together with the FV localization of PvMSP8. To confirm the protective ability of PvMSP8 antibodies, functional assays, such as a short-time growth inhibition assay, need to be developed in the future.

## Additional file

**Additional file 1: Figure S1.** Cytokine levels in supernatant of 72 hr splenocyte cultures of rPbMSP8-immunized BABL/C mice stimulated with PvMSP8 in vitro. (A) The levels (O.D. 450 nm) of splenocytes from mice immunized with rPvMSP8 after stimulation with 2.5 μg/mL of rPvMSP8. The *asterisks* indicate significant differences on the graph. (B) The levels of IFN-g, TNF-a, IL-2, IL-4, IL-10 cytokines from rPvMSP8-immunized mouse splenocytes stimulated with PvMSP8. **Table S1.** Peptide sequence information of overlapped 18-mer from the C terminus of PvMSP8. **Table S2.** Prevalence (% positive), 95% confidence intervals, and mean fluorescence intensity of IgG responses to rPvMSP8 in patient and healthy individual serum samples. **Table S3**. Prevalence, 95% confidence intervals, and normalized mean fluorescence intensity of IgG responses to rPvMSP8 in 1-month follow-up and archival sera samples.
