# Supplementary material for: Naturally acquired humoral and cellular immune responses to Plasmodium vivax merozoite surface protein 8 in patients with P. vivax infection
Source: Malar J. 2017 May 22;16:211. doi: 10.1186/s12936-017-1837-5 (PMC5440977; doi:10.1186/s12936-017-1837-5)
Supplement: Supplementary file 1 — Additional file 1: Figure S1. Cytokine levels in supernatant of 72 hr splenocyte cultures of rPbMSP8-immunized BABL/C mice stimulated with PvMSP8 in vitro. (A) The levels (O.D. 450 nm) of splenocytes from mice immunized with rPvMSP8 after stimulation with 2.5 μg/mL of rPvMSP8. The asterisks indicate significant differences on the graph. (B) The levels of IFN-g, TNF-a, IL-2, IL-4, IL-10 cytokines from rPvMSP8-immunized mouse splenocytes stimulated with PvMSP8. Table S1. Peptide sequence information of overlapped 18-mer from the C terminus of PvMSP8. Table S2. Prevalence (% positive), 95% confidence intervals, and mean fluorescence intensity of IgG responses to rPvMSP8 in patient and healthy individual serum samples. Table S3. Prevalence, 95% confidence intervals, and normalized mean fluorescence intensity of IgG responses to rPvMSP8 in 1-month follow-up and archival sera samples. [file 12936_2017_1837_MOESM1_ESM.pdf]

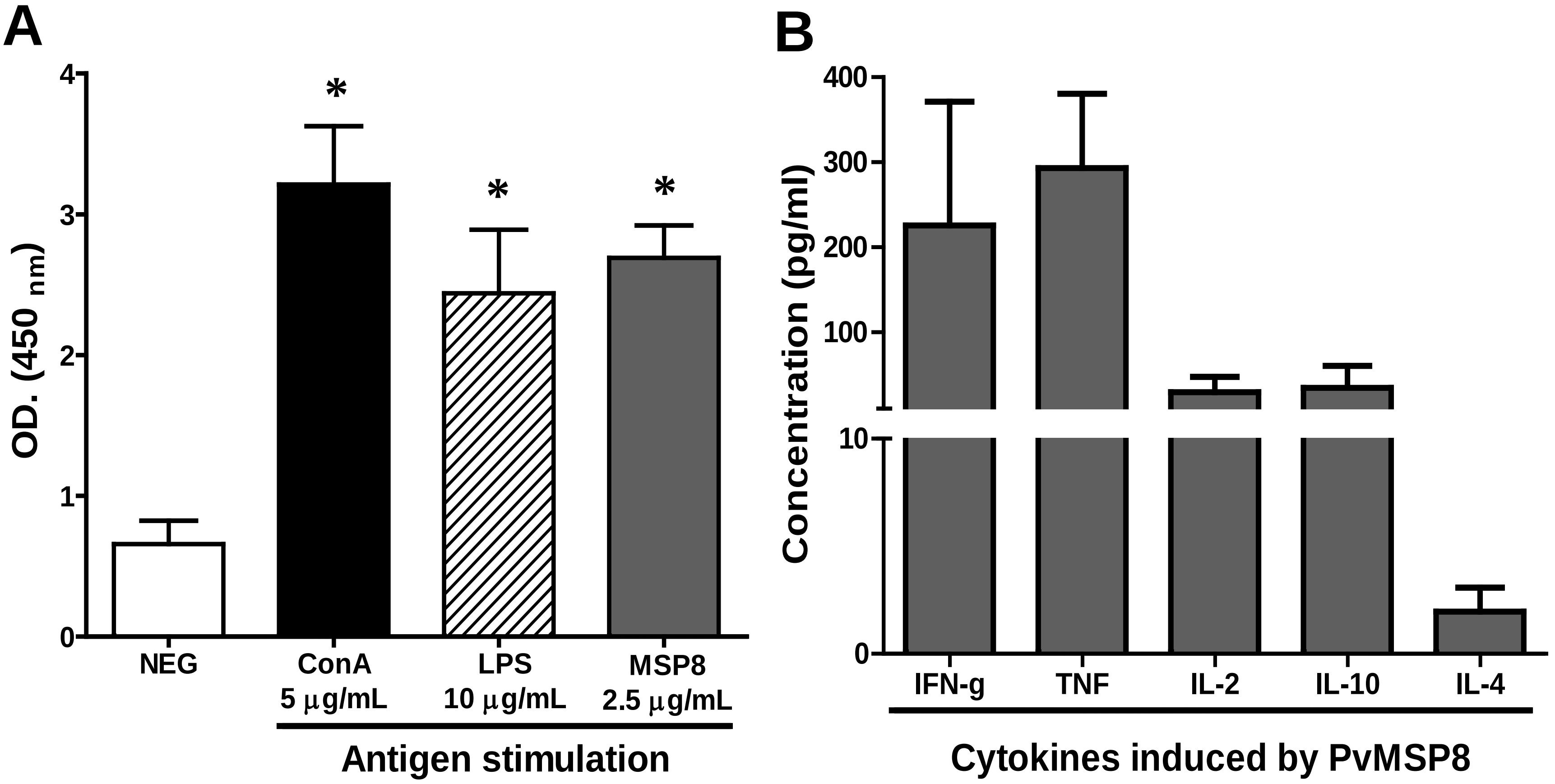
A
OD. (450 nm)
4
3
2
1
0
*
*
*
NEG
ConA
5 μg/mL
LPS
10 μg/mL
MSP8
2.5 μg/mL
Antigen stimulation
B
Concentration (pg/ml)
400
300
200
100
10
0
IFN-g
TNF
IL-2
IL-10
IL-4
Cytokines induced by PvMSP8

Suppl. Fig. 1

Table S1. Peptide sequence information of overlapped 18-mers from the PvMSP8 C terminus

No. peptide	Peptide sequence	Peptide location in full length of PvMSP8 (Sal-1)
1	EKINEHFIKDDSFDLSDY	258–275
2	DDSFDLSDYLADFELINY	267–284
3	LADFELINYIIMHETSEL	276–293
4	IIMHETSELIDELLNIIE	285–302
5	IDELLNIIESMNFRLESG	294–311
6	SMNFRLESGSLEKMVKSA	303–320
7	SLEKMVKSAESGMNLNCK	312–329
8	ESGMNLNCKMKEDIIHLL	321–338
9	MKEDIIHLLKKSSAKFFK	330–347
10	KKSSAKFFKIEIDRKTKM	339–356
11	IEIDRKTKMIYPVQATHK	348–365
12	IYPVQATHKGANMKQLAL	357–374
13	GANMKQLALSFLQKNNVC	366–383
14	SFLQKNNVCEHKKCPLNS	375–392
15	EHKKCPLNSNCYVINGEE	384–401
16	NCYVINGEEVCRCLPGFS	393–410
17	VCRCLPGFSDVKIDNVMN	402–419
18	DVKIDNVMNCVRDDTLDC	411–428
19	CVRDDTLDCSNNNGGCDV	420–437
20	SNNNGGCDVNATCTLIDK	429–446
21	NATCTLIDKKIVCECKDN	438–455
22	KIVCECKDNFEGDGIYCS	447–464

Table S2. Prevalence (% positive), 95% confidence intervals, and mean fluorescence intensity of IgG responses to rPvMSP8 in patient and healthy individual serum samples

Protein	No. of patients samples (*n*)			95%CI (%)	MFI	No. of healthy samples (*n*)			95% CI (%)	MFI	*P* value
	Positive	Negative	Total (%)			Positive	Negative	Total (%)			
MSP8	82	30	112 (73.2)	64.3-80.6	17,867	3	77	80(3.8)	89.6-98.7	1,363	< 0.0001

Abbreviations: MFI, mean fluorescence intensity; CI, confidence interval.

Table S3. Prevalence, 95% confidence intervals, and normalized mean fluorescence intensity of IgG responses to rPvMSP8 in 1-month follow-up and archival sera samples

Samples (*n*)	No. of samples		Sensitivity (%)	Specificity (%)	95% CI (%)	Normalized MFI ± SD[a]	*P* value[b]
	Positive	Negative					
Acute patients (25)	21	4	84.0		65.4-93.6	7.46 ± 1.19	< 0.0001
Day 7 (25)	25	0	100.0		86.7-100.0	8.91 ± 1.31	< 0.0001
Day 14 (25)	24	1	96.0		80.5-99.3	8.00 ± 1.08	< 0.0001
Day 21 (25)	23	2	92.0		75.0-97.8	6.84 ± 1.08	< 0.0001
Day 28 (25)	23	2	92.0		75.0-97.8	5.99 ± 0.94	< 0.0001
5 year (30)	17	13	56.7		39.2-72.6	1.582 ± 0.21	< 0.0001
10 year (30)	10	20	33.3		19.2-51.2	0.86 ± 0.08	0.002
30 year (30)	7	23	23.3		11.8-40.9	0.70 ± 0.07	0.144
Healthy (30)	0	30		100.0	88.7-100.0	0.58 ± 0.04	

Abbreviations: CI, confidence interval; SD, standard deviation.

[a] Normalized MFI, fluorescence intensities divided by each cutoff value (2 standard deviations above the mean fluorescence intensity of the malaria-naive samples).

[b] Differences in the total IgG level for each antigen between vivax malaria patients and healthy individuals were calculated with the Mann-Whitney *U* test. A *P* value of _0.05 was considered statistically significant.
